# The effect of Omicron breakthrough infection and extended BNT162b2 booster dosing on neutralization breadth against SARS-CoV-2 variants of concern

**DOI:** 10.1371/journal.ppat.1010882

**Published:** 2022-10-03

**Authors:** Carl Graham, Thomas Lechmere, Aisha Rehman, Jeffrey Seow, Ashwini Kurshan, Isabella Huettner, Thomas J. A. Maguire, Jerry C. H. Tam, Daniel Cox, Christopher Ward, Mariusz Racz, Anele Waters, Christine Mant, Michael H. Malim, Julie Fox, Katie J. Doores

**Affiliations:** 1 Department of Infectious Diseases, School of Immunology & Microbial Sciences, King’s College London, London, United Kingdom; 2 Harrison Wing, Guys and St Thomas’ NHS Trust, London, United Kingdom; 3 Infectious Diseases Biobank, Department of Infectious Diseases, School of Immunology and Microbial Sciences, King’s College London, London, United Kingdom; NYU School of Medicine, UNITED STATES

## Abstract

COVID-19 vaccines are playing a vital role in controlling the COVID-19 pandemic. As SARS-CoV-2 variants encoding mutations in the surface glycoprotein, Spike, continue to emerge, there is increased need to identify immunogens and vaccination regimens that provide the broadest and most durable immune responses. We compared the magnitude and breadth of the neutralizing antibody response, as well as levels of Spike-reactive memory B cells, in individuals receiving a second dose of BNT162b2 at a short (3–4 week) or extended interval (8–12 weeks) and following a third vaccination approximately 6–8 months later. We show that whilst an extended interval between the first two vaccinations can greatly increase the breadth of the immune response and generate a higher proportion of Spike reactive memory B cells, a third vaccination leads to similar levels between the two groups. Furthermore, we show that the third vaccine dose enhances neutralization activity against omicron lineage members BA.1, BA.2 and BA.4/BA.5 and this is further increased following breakthrough infection during the UK omicron wave. These findings are relevant for vaccination strategies in populations where COVID-19 vaccine coverage remains low.

## Author summary

COVID-19 vaccines have been vital in controlling the current pandemic. With the emergence of SARS-CoV-2 viral variants, it is important to understand factors that influence the neutralization breadth of vaccine responses. Here we study the impact of the interval between the 1^st^ and 2^nd^ BNT162b2 vaccine dose on neutralization breadth and how this is further affected by vaccine boosters and breakthrough infections.

## Introduction

Development of vaccines against SARS-CoV-2 has been a vital step in controlling the global COVID-19 pandemic. Most approved vaccines use the SARS-CoV-2 Spike antigen to elicit a neutralizing antibody response as well as generating cell-mediated immunity. The Spike glycoprotein interacts with the angiotensin-converting enzyme 2 (ACE2) on host cells and facilitates viral entry. One of the greatest challenges faced by current vaccines has been the emergence of SARS-CoV-2 viral variants of concern (VOCs) that encode mutations in the Spike protein, including alpha (B.1.1.7), beta (B.1.351), delta (B.1.617.2) and omicron (sub-lineage members BA.1, BA.2, BA.4 and BA.5). Numerous studies have shown that these Spike mutations can lead to partial escape in convalescent and vaccinee sera, with the greatest reduction in neutralization being observed for omicron sub-lineages [1–5]. Therefore, immunogens and immunization regimes that elicit durable neutralizing antibody (nAb) responses with broad activity against both known and newly emerging variants is highly desirable.

In December 2020, the Pfizer/BioNTech BNT162b2 mRNA COVID-19 vaccine was given approval for use in the UK. The approved regimen was two doses administered at a 3–4-week interval, but in January 2021 a change to UK policy meant that the BNT162b2 booster vaccination was administered with an extended interval of 8–12 weeks. The rationale behind this change was to provide as much of the UK population with some level of immunity in the face of a large wave of SARS-CoV-2 alpha variant infections [6]. Initially, most COVID-19 vaccines required two vaccinations to provide efficacy against the Wuhan (wild-type, WT) alpha and delta VOCs. However, due to waning of vaccine-elicited nAb levels [7] and with the emergence of omicron/BA.1, which encodes >30 mutations in Spike, a third vaccination (either BNT162b2 or mRNA-1273) became recommended. A third vaccine has been shown to increase neutralization titres against omicron/BA.1 [3–5,8] and to restore vaccine efficacy against omicron/BA.1 [9–11]. However, breakthrough infections (BTI) in vaccinated individuals have been observed at increased frequencies compared to previous dominant variants [9–11].

We sought to determine how the interval between the 1^st^ and 2^nd^ BNT162b2 vaccinations impacted on neutralization breadth and potency against current and newly emerging VOCs in the short-term (post 2-doses) and in the long-term (post 3-doses), and how subsequent BTI during the UK omicron wave (January to February 2022) affected neutralization of newer omicron sub-lineage members. We show that broader plasma neutralizing activity is observed when the 2^nd^ dose is given with extended 8–12 week interval (extended group) compared to those receiving the 2^nd^ dose with a 3–4 week interval (short group). However, the advantage of the extended booster to neutralization is not displayed following a 3^rd^ vaccination where robust neutralization of omicron lineages members (BA.1, BA.2, BA.4 and BA.5) is observed in both groups. Finally, we show that omicron BTI further boosts the neutralization activity against omicron sub-lineages. Overall, this research provides insights into optimizing vaccine regimens to provide the greatest neutralization breadth.

## Results

### Vaccine cohort description

**Fig 1:**
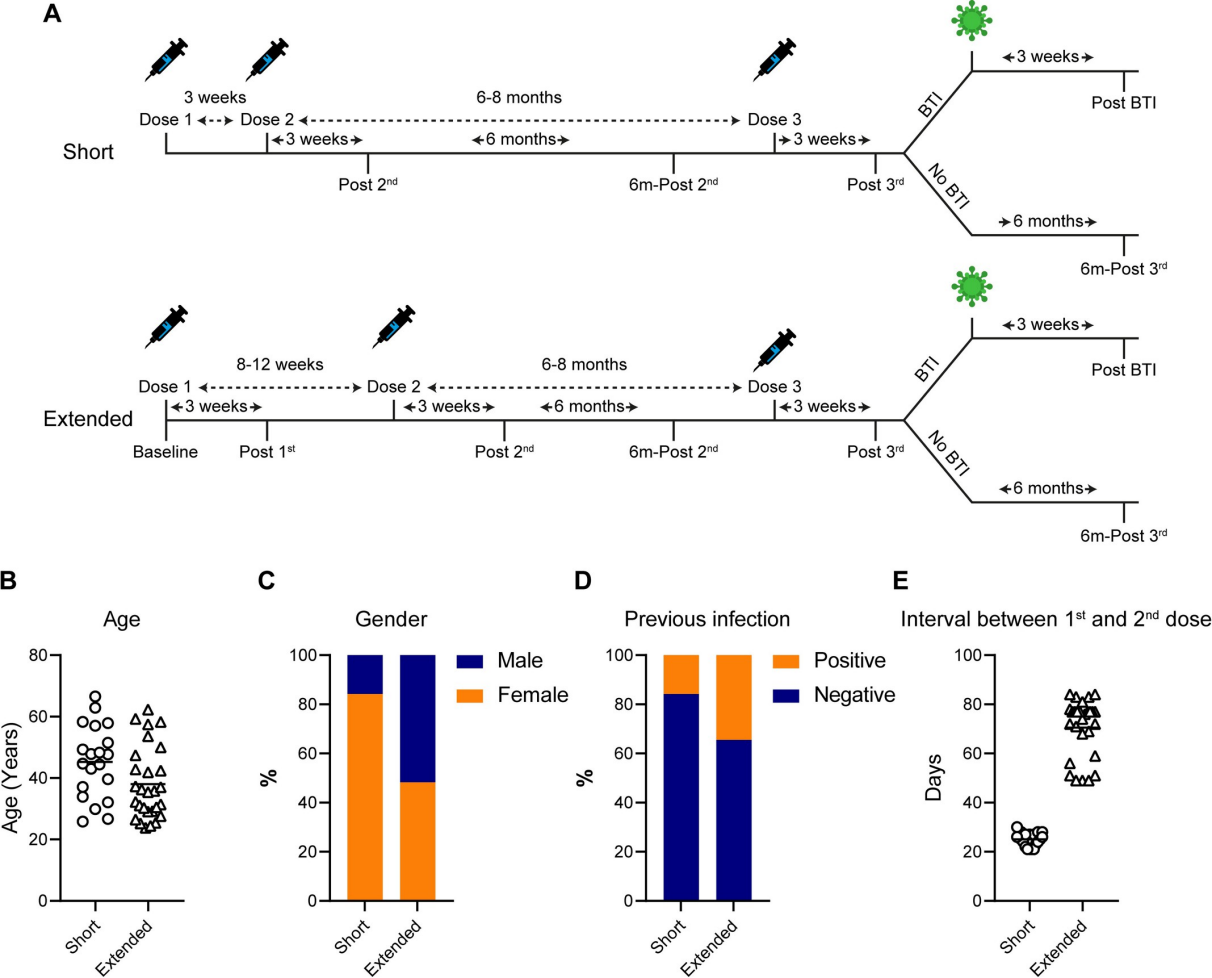
**A)** Vaccination and blood sampling schedule in short and extended vaccine groups. Horizontal line shows the mean age for each group. **B)** Difference in age between short- and extended-groups. **C)** Gender of participants in each group. **D)** Frequency of previous SARS-CoV-2 infection. **E)** Length of time between the 1^st^ and 2^nd^ dose of BNT162b2 vaccine. Horizontal line shows the mean days between vaccine doses. Short- and extended-groups shown in circle and triangles, respectively.

Plasma and PBMCs were collected from individuals receiving the BNT162b2 vaccine either with a short booster interval (3–4 weeks, n = 19, short-group) or an extended booster interval (8–12 weeks, n = 28, extended-group) (**Fig 1A**). For the extended-group blood was collected prior to vaccination (baseline), 3 weeks post 1^st^ dose (post 1^st^) and post 2^nd^ dose (post 2^nd^). For the short-group, blood was only collected post 2^nd^ dose (post 2^nd^). Blood was also collected from both groups 6 months post 2^nd^ vaccine dose (6m-post 2^nd^) and further samples was taken 3–4 weeks and 6-months post 3^rd^ vaccine (post 3^rd^ and 6m-post 3^rd^, respectively). For individuals experiencing a BTI, blood was also collected post infection (post BTI).

The short-group was 84.2% female, with an average age of 44.1 years (standard deviation, 11.1 years) whereas the extended-group was 53.6% female, with a slightly lower average age of 38.0 years (standard deviation, 11.8 years) (**Fig 1B and 1C**). Information on previous SARS-CoV-2 infection was recorded at each blood draw. In addition, as utilised previously [12,13], the presence of IgG to nucleoprotein and Spike was utilized to determine previous asymptomatic SARS-CoV-2 infections or infections occurring prior to community PCR testing in those with a baseline sample (extended-group). For those without a baseline sample, the presence of IgG to nucleoprotein post 2^nd^ dose was used. Previous infection was reported and/or detected in 3/19 from the short-group and in 10/28 from the extended-group (**Fig 1D**), and SARS-CoV-2 naïve individuals were grouped separately for analysis. All participants reporting infection experienced mild symptoms that did not require hospitalization. Based on the reported timing of infection (February to October 2020), the majority of the infections were presumed to be with the ancestral strain. However, two participants in the extended-group reported infections in December 2020 when alpha was becoming dominant in the UK.

The median interval between vaccine doses was 26 days (interquartile range, 3.5 days) for the short group and 77.0 days (interquartile range, 11.3 days) in the extended group (**Fig 1E**). Participants in the short-group received their COVID-19 vaccine earlier due to their roles as front-line healthcare workers rather than being deemed at higher risk due to co-morbidities. One participant in the short-group was Type-2 diabetic and one participant in the extended-group was receiving treatment for rheumatoid arthritis and the remaining donors were otherwise considered healthy.

### Anti-Spike IgG levels are similar in short and extended booster groups

**Fig 2:**
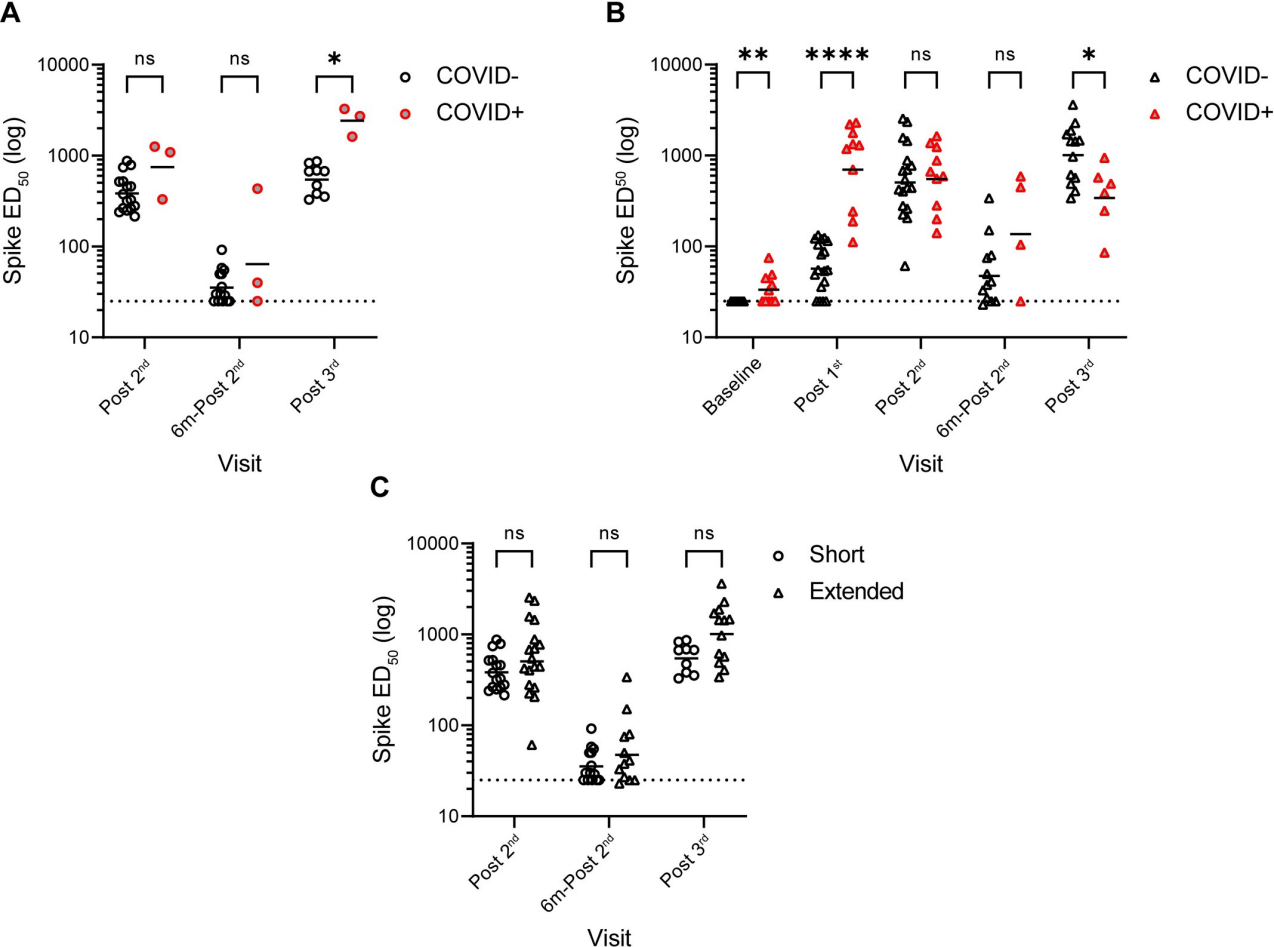
IgG binding titres to SARS-CoV-2 Spike over time. **A**) Comparison of geometric mean ED_50_ values between SARS-CoV-2 naïve individuals (open black circle) and those previously infected before vaccination (grey/red circles) in short-booster group. **B**) Comparison of geometric mean ED_50_ values between SARS-CoV-2 naïve individuals (open black triangle) and those previously infected before vaccination (grey/red triangle) in extended-booster group. **C**) Comparison of geometric mean ED_50_ values between short- and extended-booster groups post 2^nd^ and 6 months post 2^nd^ and post 3^rd^ vaccine. Dotted line represents the lowest limit of detection for the assay. D’Agostino and Pearson tests were performed to determine normality. Based on this result, multiple Mann-Whitney tests or unpaired t tests using a two-stage linear step-up procedure of Benjamini, Krieger and Yekutieli were employed to determine significance between groups. ns P > 0.05, * P ≤ 0.05, ** P ≤ 0.01, *** P ≤ 0.001 and **** P ≤ 0.0001.

IgG binding to recombinant Wuhan-1 Spike was measured by ELISA and the half-maximal binding (ED_50_) calculated (**Fig 2**). As reported previously [14–18], individuals who had experienced a SARS-CoV-2 infection prior to vaccination had higher Spike IgG levels compared to naïve individuals after the 1^st^ vaccine dose (post 1^st^) (**Fig 2B**). However, similar levels of anti-Spike IgG were observed in the SARS-CoV-2 naïve and convalescent donors following the 2^nd^ vaccination (post 2^nd^) (**Fig 2A** and **2B**).

For SARS-CoV-2 naïve individuals, comparison of ED_50_ between the short and extended groups showed no statistical differences post 2^nd^, although there was a trend towards higher levels in the extended group (**Fig 2C**). Both groups showed waning of the anti-Spike IgG levels 6 months post 2^nd^ vaccine (6m-post 2^nd^) which were subsequently boosted after participants received a 3^rd^ vaccine dose (post 3^rd^) (**Fig 2A and 2B**).

### Previous SARS-CoV-2 infection leads to higher nAb titres following the first vaccine dose

**Fig 3:**
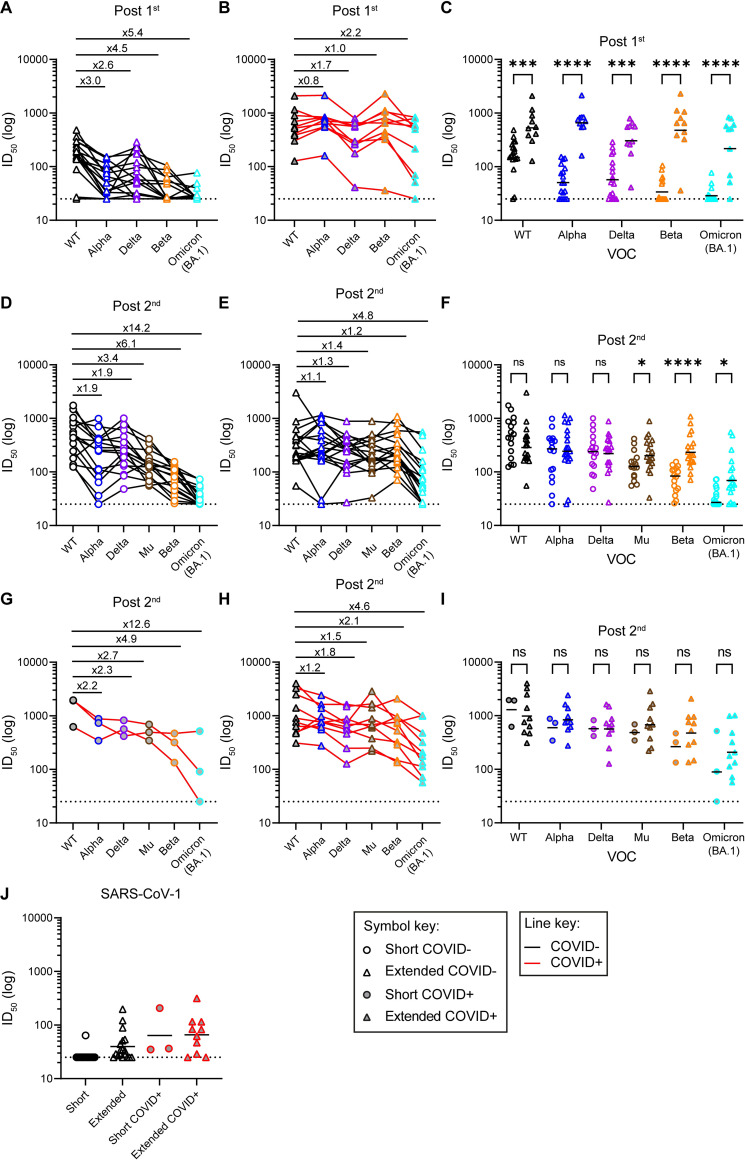
Neutralization breadth and potency following two-doses of BNT162b2. Neutralization was measured against wuhan-1 (WT), alpha, delta, mu, beta and omicron/BA.1. **A)** Neutralization following a single BNT162b2 vaccine dose (post 1^st^) in naïve individuals (n = 18). **B)** Neutralization following a single BNT162b2 vaccine dose in individuals infected with SARS-CoV-2 prior to vaccination (n = 10). Data from individual donors are linked and fold changes compared to WT shown above. **C)** GMT comparison between SARS-CoV-2 naïve individuals (open triangle) and convalescent donors (grey triangle) following one vaccine dose. The horizontal line shows the GMT. Neutralization data following 2-vaccine doses (post 2^nd^) in SARS-CoV-2 naïve individuals in **D)** the short booster group (n = 16), and **E)** the extended booster group (n = 18). Data from individual donors are linked and fold changes compared to WT shown above. **F)** GMT comparison in SARS-CoV-2 naïve individuals receiving the short booster interval (open circle) and extended booster (open triangle). The horizontal line shows the GMT. Neutralization data following 2-vaccine doses (post 2^nd^) in SARS-CoV-2 convalescent individuals in **G)** the short booster group (n = 3) and **H)** the extended booster group (n = 10). Data from individual donors are linked and fold changes compared to WT shown above. **I)** GMT comparison in SARS-CoV-2 convalescent individuals receiving the short booster interval (grey circle) and extended booster (grey triangle). The horizontal line shows the GMT. **J)** Neutralization of SARS-CoV-1. The horizontal dotted line is the lowest limit of detection. D’Agostino and Pearson tests were performed to determine normality. Based on this result, multiple Mann-Whitney tests using a two-stage linear step-up procedure of Benjamini, Krieger and Yekutieli were employed to determine significance between groups. ns P > 0.05, * P ≤ 0.05, ** P ≤ 0.01, *** P ≤ 0.001 and **** P ≤ 0.0001.

Next, plasma neutralization breadth and potency were determined using HIV-1 lentiviral particles pseudotyped with either the SARS-CoV-2 Spike of Wuhan-1 (vaccine strain), alpha, delta, beta or omicron/BA.1 VOCs and a HeLa cell line stably expressing ACE2 as the target cell [13]. We have previously shown that the ID_50_ values generated using the pseudotype assay correlate well with ID_50_ values obtained using a live virus neutralization assay [13,19]. Analysis of neutralizing responses after a single vaccine dose (post 1^st^) was only conducted on the extended-group due to sample availability (**Fig 1A**). Consistent with previous studies [14–18], following 1-dose of BNT162b2, geometric mean titres (GMTs) against the matched vaccine strain (Wuhan-1, wildtype, WT) were higher is those who had a SARS-CoV-2 infection prior to vaccination (**Fig 3A–3C**). Furthermore, previously infected individuals showed greater neutralization breadth against VOCs (**Fig 3A–3C**).

### An extended interval between the 2^nd^ vaccine dose enhances the breadth of the neutralizing antibody response

Following two doses of BNT162b2 in SARS-CoV-2 naïve individuals, GMTs against the matched vaccine strain (Wuhan-1, WT) were similar between the short and extended booster groups (**Fig 3D–3F**). In both the short and extended-groups, higher GMTs were observed for individuals that had a SARS-CoV-2 infection prior to vaccination (**Fig 3G–3I**) [14–18]. In SARS-CoV-2 naïve individuals (**Fig 3D and 3E**), the broadest neutralization activity against VOCs was observed in the extended booster group where a modest 1.1–1.4-fold reduction in neutralization was observed against alpha, delta, mu and beta and a 4.8-fold reduction against omicron/BA.1 (**Fig 3E**). For those in the short-group, a larger reduction in neutralization potency against VOCs was observed, with greater reductions against beta (6.1-fold) and omicron (14.2-fold) (**Fig 3D**). Comparison between GMTs of the short and extended booster groups showed a significantly reduced potency against mu, beta and omicron/BA.1 VOCs in the short booster group (**Fig 3F**). Although overall GMTs against VOCs were higher in previously infected vaccinees (**Fig 3G–3I**), the extended booster group also showed higher GMTs against beta and omicron/BA.1 than the short booster group. However, this difference did not reach significance due to the small sample size (**Fig 3I**).

To determine whether the extended booster generated neutralization breadth beyond SARS-CoV-2 VOCs, neutralization was also measured against SARS-CoV-1 which shares 73% sequence [20] similarity with Spike of SARS-CoV-2. Although neutralization titres were generally low, individuals receiving the extended booster showed low levels of SARS-CoV-1 neutralization (**Fig 3J**) with a GMT that was higher than in the short-group.

### An extended vaccine interval enhances the magnitude of the memory B cell response

**Fig 4:**
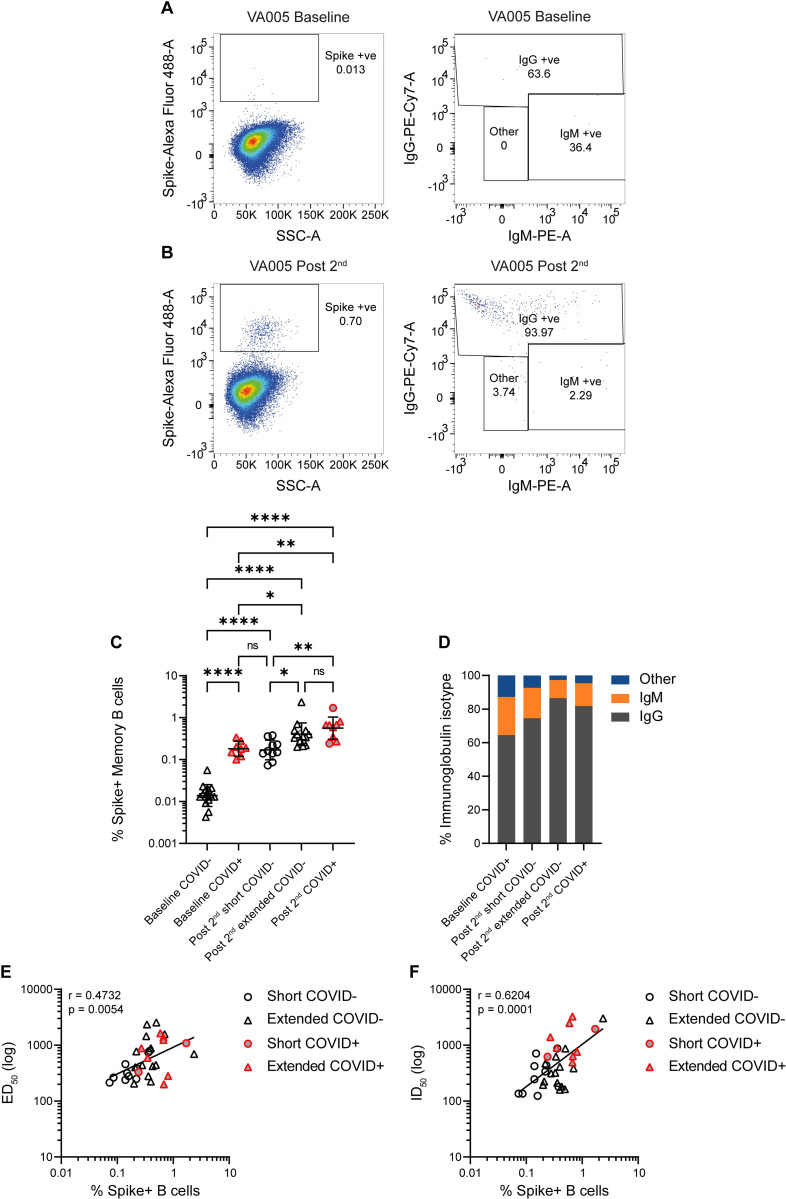
Higher percentage of SARS-CoV-2 Spike-reactive memory B cells are detected in the extended booster group. Percentage of Spike-reactive memory B cells were determined by flow cytometry. **A)** Example pre-bleed (baseline) PBMC staining for SARS-CoV-2 naïve individual. **B)** Example post 2^nd^ vaccine PBMC staining for SARS-CoV-2 naïve individual. Example of full flow analysis shown if **S1A Fig**. **C)** Percentage Spike-reactive memory B cells at baseline and post 2^nd^ vaccine were determined by flow cytometry. Previously infected individuals shown in red/grey. Extended and short interval groups shown with triangle and circle symbols, respectively. D’Agostino and Pearson tests were performed to determine normality. Based on this result, differences between groups were assessed using a Brown-Forsythe ANOVA test with Dunnett’s T3 multiple comparisons post hoc. ns P > 0.05, * P ≤ 0.05, ** P ≤ 0.01, *** P ≤ 0.001 and **** P ≤ 0.0001. **D)** Percentage of IgG, IgM or other Spike-reactive memory B cells for each vaccine group. **E)** Correlation between Spike IgG ED_50_ and percentage of Spike-reactive memory B cells. **F)** Correlation between ID_50_ against Wuhan-1 pseudotyped virus and percentage of Spike-reactive memory B cells. (Spearman’s correlation, r; a linear regression was used to calculate the goodness of fit, r^2^).

To further examine how vaccine interval impacts on the B cell response, we next measured the frequencies of Spike-reactive memory B cells in the short and extended booster groups using flow cytometry (**Figs 4A, 4B** and **S1A**) [14,21]. The frequency of Wuhan-1 Spike-reactive memory B cells was measured in pre-vaccination samples (baseline) for the extended booster group only (due to sample availability) and in both groups following the 2^nd^ vaccination (post 2^nd^). Overall, where paired samples were available, the overall frequency of memory B cells did not change over the course of the analysis (**S1B Fig**). Participants who had a SARS-CoV-2 infection prior to vaccination had a distinct population of Spike reactive memory B cells in the pre-vaccine baseline sample (mean 0.20%, range 0.10–0.34%) compared to naïve individuals (mean 0.02%, range 0.01–0.06%) (**Fig 4C**). Following two vaccine doses, the percentage of Spike-reactive memory B cells increased independent of prior SARS-CoV-2 exposure (**Fig 4C**).

In SARS-CoV-2 naïve participants, a higher frequency of Spike reactive memory B cells was observed in the extended booster group (mean 0.52%, range 0.20–2.36%) compared to the short booster group (mean 0.19%, range 0.07–0.38%) after the 2^nd^ vaccine dose (**Fig 4C**). When considering the frequency of Spike-reactive B cells in previously infected vaccinated donors, the percentage (mean 0.67%, range 0.24–1.71%) was similar to SARS-CoV-2 naïve individuals in the extended group.

The isotype of the Spike-reactive memory B cells was considered further (**Fig 4D**). In samples collected from previously infected individuals prior to vaccination, an average 64.5% of the Spike-reactive memory B cells were IgG positive (**Fig 4D**) and the average percentage increased upon vaccination to 81.8% (**Fig 4D**) suggesting an improvement in the quality of the response. In COVID-19 naïve individuals, a higher percentage of IgG+ Spike-reactive memory B cells was seen in the extended-booster group (86.5%) compared to the short-booster group (74.5%). A positive correlation between the percentage of Spike-reactive memory B cells and the ED_50_ and ID_50_ (**Fig 4E and 4F**) was observed after two vaccine doses independent of booster interval or previous SARS-CoV-2 exposure.

Overall, an extended period between the first and second vaccine dose increases the magnitude of the Spike-specific B cell response.

### Neutralizing antibody levels decline at 6 months but are boosted and neutralization broadened by a third vaccine dose

**Fig 5:**
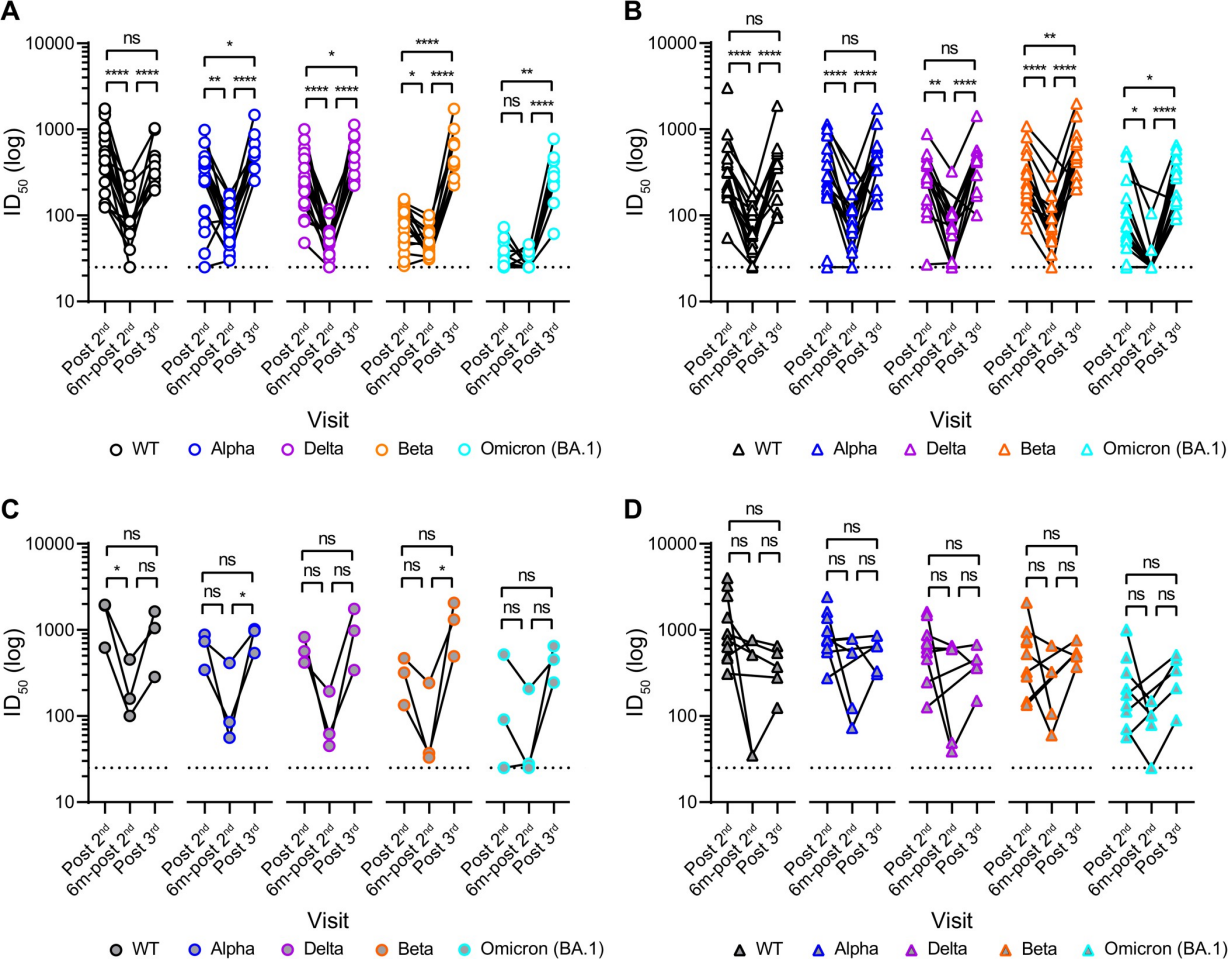
Neutralization potency decreases 6-months post 2^nd^ vaccine but breadth and potency increase after a 3^rd^ vaccine dose. Neutralization ID_50_ against WT, alpha, delta, beta and omicron/BA.1 post 2^nd^, 6 months post 2^nd^ and post 3^rd^ vaccine for **A)** SARS-CoV-2 naïve individuals in the short-group (circle) and **B)** SARS-CoV-2 naïve individuals in the extended-grous (triangle) and **C)** previously infected individuals in the short-group (grey circle) and **D)** previously infected individuals in the extended-group (grey triangle). Difference between GMT post 2^nd^, 6 months post 2^nd^ and post 3^rd^ vaccine were determined using Mann-Whitney unpaired t-test. D’Agostino and Pearson tests were performed to determine normality. Based on this result, differences between groups were assessed using either one-way ANOVA (Tukey’s multiple comparisons post hoc), Kruskal-Wallis test (Dunn’s multiple comparisons post hoc) or Friedman test (Dunn’s multiple comparisons post hoc). ns P > 0.05, * P ≤ 0.05, ** P ≤ 0.01, *** P ≤ 0.001 and **** P ≤ 0.0001.

To understand the durability of the nAb response following two vaccine doses, neutralization titres were measured 6 months post second vaccine dose (6m-post 2^nd^). Plasma neutralization activity against all VOCs (WT, alpha, delta, beta and omicron (BA.1)) had declined in both groups (**Fig 5A–5D**) as reported in other recent studies [4,8,22]. Although the neutralization of the most antigenically distant VOCs (beta and omicron/BA.1) had declined, titres remained higher in the extended booster group compared to the short booster group (**Fig 5A** and **5B**).

In September 2021, a vaccine booster programme was initiated in the UK and blood was collected from individuals 3-weeks after receiving a 3^rd^ vaccine dose (post 3^rd^). The impact on IgG levels to Spike and neutralization breadth and potency was measured. The third vaccine dose (post 3^rd^) increased Spike IgG binding **(Fig 2)** and the plasma neutralizing activity (**Fig 5A–5D**) compared to 6 months post 2^nd^ vaccine in both groups. Whereas after the 2^nd^ dose there had been a clear distinction in neutralization breadth between the short and extended booster groups, following administration of a third vaccine dose the neutralizing titres against WT, alpha, delta, beta and omicron/BA.1 were very similar (**Fig 5A–5D**). Importantly, a 3^rd^ vaccination with ancestral SARS-CoV-2 spike increased neutralization breadth against the most highly diverse beta and omicron/BA.1 variants (**Fig 5A–5D**). Overall, the reduced neutralization breadth observed in the short booster group after two vaccine doses is rescued following a third vaccine dose.

### Broader neutralization is associated with have higher avidity against Spike

**Fig 6:**
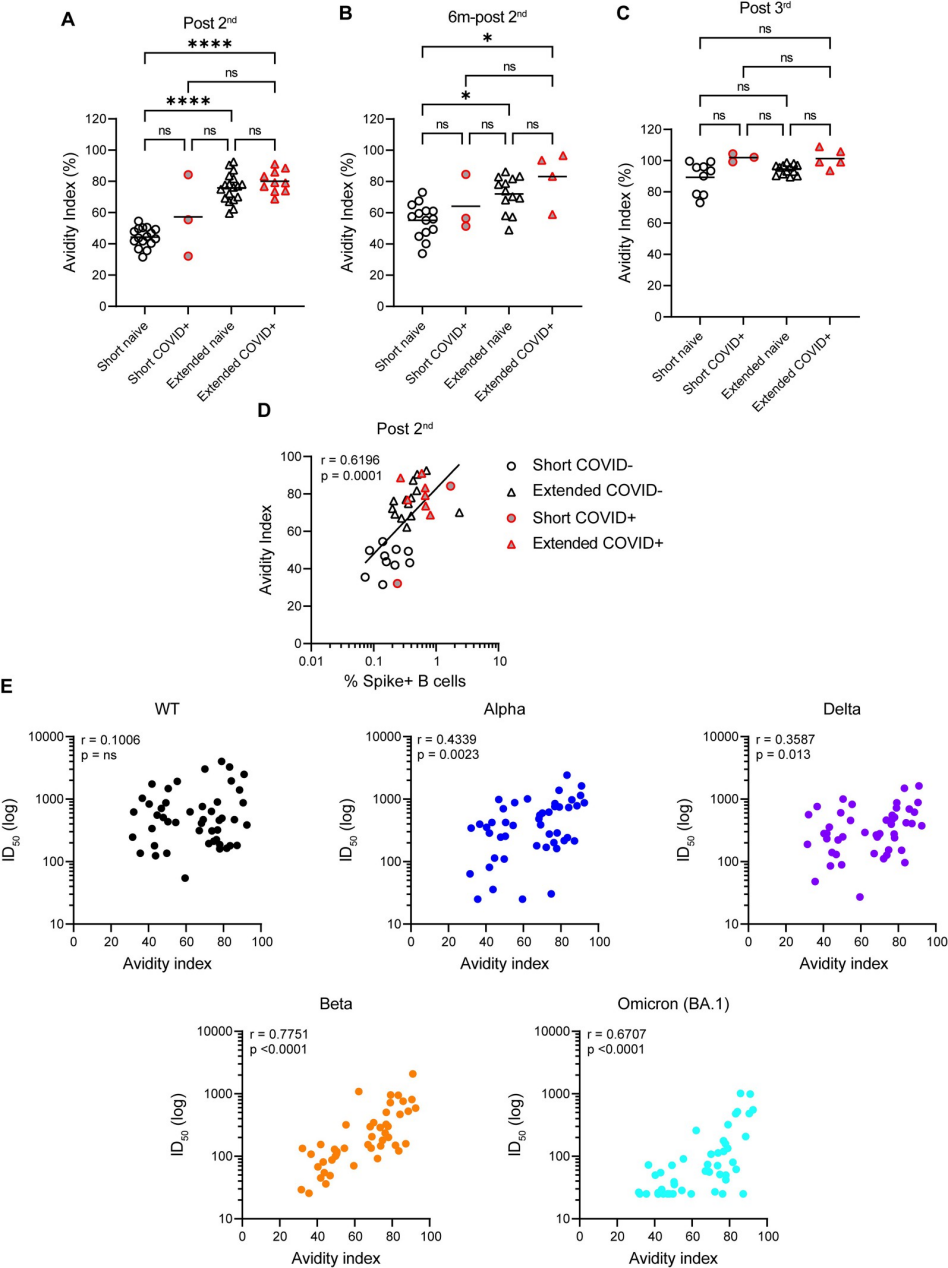
IgG binding avidity increases with vaccine boosting. Avidity index for IgG binding to Spike was measured **A)** post 2^nd^, **B)** 6 months post 2^nd^ and **C)** post 3^rd^ and compared across groups. Avidity index was calculated by comparing the area under the curve (AUC) with/without an 8M Urea washing step. D’Agostino and Pearson tests were performed to determine normality. Based on this result, differences between groups were assessed using a Kruskal-Wallis test with Dunn’s multiple comparisons post hoc. **D)** Correlation between avidity index and % Spike-reactive memory B cells. (Spearman’s correlation, *r*; a linear regression was used to calculate the goodness of fit, *r*^*2*^). **E)** Correlations between avidity index and ID_50_ against VOCs following 2-doses of BNT162b2 vaccine.

Antibody binding avidity has been associated with antibody maturation [23–27]. To determine whether enhanced neutralization breadth was related to higher avidity of antibody binding in the extended booster group we next measured an avidity index for samples collected 3-weeks and 6 months after the second vaccine dose (post 2^nd^ and 6m-post 2^nd^) and 3-weeks after the third vaccine dose (post 3^rd^). The avidity was measured by comparing the area under the curve in ELISA, with and without an 8M Urea washing step. For SARS-CoV-2 naïve individuals post 2^nd^ dose, the extended booster group had a significantly higher avidity index than the short booster group (**Fig 6A**). However, this difference was reduced when comparing previously infected individuals. The avidity index 6 months post 2^nd^ vaccine (6m-post 2^nd^) was largely unchanged (**Fig 6B**). Following the 3^rd^ vaccination (post-3^rd^) where neutralization breadth was similar across the two groups, the avidity index increased in both groups and the difference in avidity index between the short and extended groups was no longer significant (**Fig 6C**). The avidity index 3-weeks after 2^nd^ vaccine dose (post 2^nd^) was correlated with the ID_50_ against each VOC (**Fig 6E**). No significant correlation was observed for WT. However, the correlation coefficient was greatest for beta and omicron/BA.1 which are most antigenically distant to the vaccine strain and suggests that avidity may be a good indicator VOC cross-reactivity. The avidity index also correlated with the % of Spike-reactive memory B cells (**Fig 6D**).

### Neutralization of BA.2 and BA.4/5 sub-lineages

Since omicron/BA.1 became the dominant global variant worldwide, several related SARS-CoV-2 omicron sub-lineages have been reported that encode unique amino acid changes in Spike. BA.2 has driven a recent wave in the UK and encodes 21 identical mutations to BA.1 (including K417N, N440K and E484A) and 8 additional mutations across NTD and RBD. BA.4 and BA.5, which encode identical Spikes proteins, are thought to be driving a 5^th^ wave in South Africa [28]. The BA.4/BA.5 Spike is similar to BA.2 but has additional mutations delta69-70, L452R and F486V and lacks the Q493R mutation. Emergence of these highly transmissible sub-lineages has led to an increased rate of BTIs and, within the cohort described here, thirteen participants (6 and 7 in the short- and extended-groups, respectively) experienced BTI between mid-December 2021-March 2022. The average time between 3^rd^ vaccine dose and BTI was 141 days (standard deviation 39 days) and 52 days (standard deviation 24 days) for the short- and extended-groups, respectively. In mid-December BA.1 made up >80% of UK infections and the BA.1 and BA.2 sub-lineages made up 95% of infections from mid-January [29]. Therefore, BTIs in participants were presumed to be caused by BA.1 or BA.2 VOCs (post-BTI). Blood was also collected from six participants 6 months post 3^rd^ dose (6m-post 3^rd^).

**Fig 7:**
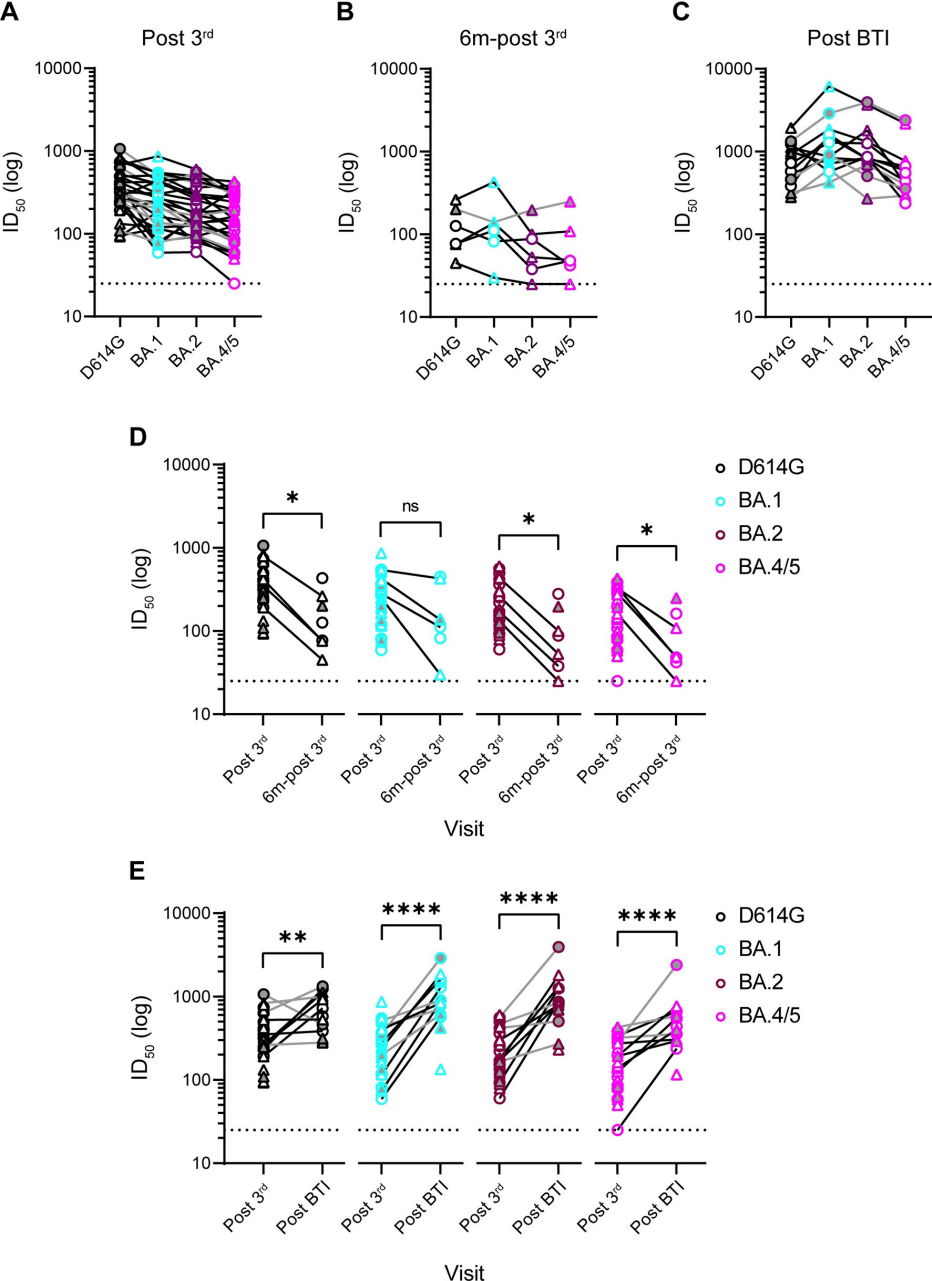
Neutralization against omicron sub-lineages following 3 vaccine doses and/or BA.1/BA.2 breakthrough infection. Neutralization breadth and potency was measured against D614G, BA.1, BA.2 and BA.4/BA.5. Analysis of sera collected **A)** 3-weeks post 3^rd^ BNT162b2 vaccine dose (post 3^rd^), **B)** 6-months post 3^rd^ vaccine (6m-post 3^rd^) and **C)** following breakthrough infection (post-BTI) (presumed to be BA.1 or BA.2). **D)** Comparison of titres 3-weeks (post 3^rd^) and 6-months post 3^rd^ boost (6m-post 3^rd^). **E)** Comparison of titres 3-weeks post 3^rd^ boost (post 3^rd^) and following BTI (post-BTI). Samples from a single individual are joined. Short and long booster groups shown with circle and triangle symbols, respectively. Individuals infected with SARS-CoV-2 prior to vaccination are shaded grey. Based on this result, multiple Mann-Whitney tests or unpaired t tests using a two-stage linear step-up procedure of Benjamini, Krieger and Yekutieli were employed to determine significance between groups. ns P > 0.05, * P ≤ 0.05, ** P ≤ 0.01, *** P ≤ 0.001 and **** P ≤ 0.0001.

To assess susceptibility to the recently reported BA.4/BA.5 variants and to determine the impact of BA.1/BA.2 BTI on the antibody response, we measured neutralization against D614G, BA.1, BA.2 and BA.4/BA.5 following three BNT162b2 doses (at 3-weeks and 6-months post 2^nd^ dose) and in those who subsequently experienced a BTI (3-weeks post-BTI) (**Fig 7**). Due to the smaller number of samples available, all plasma were considered together for analysis. For plasma collected 3-weeks post 3^rd^ vaccine dose (post-3^rd^), good neutralization against BA.1, BA.2 and BA.4/5 was detected but GMTs were slightly decreased compared to D614G, with the greatest reduction being 2.2-fold against BA.4/5 (**Fig 7A**). Six months post 3^rd^ vaccine dose (6m-post 3^rd^), neutralization titres had decreased against all variants (**Fig 7B** and **7D**) as seen six months post 2^nd^ vaccine dose (**Fig 5**). However, in those who experienced a BA.1/BA.2 BTI (post-BTI) neutralization titres increased against all variants compared to post 3^rd^ vaccine and robust cross-neutralization of all omicron sub-lineages was detected (**Fig 7C** and **7E**). The largest fold increase in GMT following BTI was observed against BA.1 and BA.2 (**Fig 7E**).

Overall, a third vaccine dose generates neutralization against omicron sub-lineages BA.1, BA.2 and BA.4/5 which is further enhanced by BA.1/BA.2 BTI.

## Discussion

Elicitation of nAbs with broad activity against both known and newly emerging variants is highly desirable and vital in controlling the current COVID-19 pandemic. Here we studied the impact of vaccine interval on the breadth and potency of the SARS-CoV-2 neutralizing antibody response. We showed that an extended interval between the 1^st^ and 2^nd^ dose of the BNT162b2 vaccine enhances neutralization breadth initially. However, a third vaccine dose generated a robust and broad neutralizing response against VOCs independent of the interval between 1^st^ and 2^nd^ vaccine doses. It has been extensively reported that ‘hybrid’ immunity, immunity derived from SARS-CoV-2 infection followed by vaccination, leads to superior neutralization following 1-dose of COVID-19 vaccine [14–18]. Our results showing higher antibody binding and neutralization in previously infected individuals receiving one BNT162b2 dose are consistent with these reports.

Here, we show than an extended interval between the 1^st^ and 2^nd^ vaccine dose enhances the immune response in four ways. Firstly, and similar to previous reports, there is an increase in IgG binding (ED_50_) to Spike in the extended booster group following the 2^nd^ immunization [25,30,31] and secondly, the Spike-reactive IgG bind with higher avidity [25]. Thirdly, broader neutralizing activity against VOCs (including BA.1) is generated [32,33]. Finally, a higher percentage of Spike-reactive IgG^+^ memory B cells is detected in SARS-CoV-2 naïve individuals receiving an extended booster. Binding avidity correlated most strongly with the ID_50_ against the most antigenically distinct VOCs. The higher binding avidity in the extended group suggests that antibodies have undergone higher levels of antibody maturation [23–26]. Indeed, wider SARS-CoV-2 neutralization breadth has been associated with increased somatic hypermutation [14,34–36]. We have recently reported that mAbs isolated from an individual receiving 2-doses of the ChadOx1 AZD1222 vaccine at a 12-week interval were more highly mutated than those isolated from SARS-CoV-2 convalescent donors and displayed greater neutralization breadth [34]. Several studies have shown that mRNA vaccination leads to a robust memory B cell response and induces persistent germinal centre reactions that continue for months after primary vaccination [14,21,37,38]. Therefore, a longer interval between the 1^st^ and 2^nd^ vaccine dose would allow extended germinal centre reactions to take place, leading to increased levels of somatic hypermutation and higher affinity to vaccine antigen.

Neutralization titres decreased 6 months post 2^nd^ vaccine dose [22]. However, consistent with previous research, we show that titres are boosted following the 3^rd^ BNT162b2 vaccination. Importantly, neutralization breadth increased against beta and omicron sub-lineages independent of the interval between the 1^st^ and 2^nd^ vaccine doses leading to overall similar levels of cross-neutralizing activity in both the short and extended booster groups. This increase in neutralization breadth was accompanied by an increase in the Spike-reactive IgG binding avidity in the short booster group, indicative of continued affinity maturation. Nussenzweig and co-workers identified antibody lineages that had undergone further somatic hypermutation following a 3^rd^ dose of COVID-19 mRNA vaccine and these mAbs had superior neutralization breadth and potency [39]. Relevant to the COVID-19 pandemic at the time of writing (June 2022), neutralization against the antigenically distant VOCs BA.1 [3–5,8], BA.2 [2,40,41] and BA.4/BA.5 [42–44] also increased following a 3^rd^ vaccine dose in both groups.

Following BTI during the UK BA.1 or BA.2 wave, neutralization titres against D614G, BA.1, BA.2 and BA.4/BA.5 increased in this cohort independent of previous SARS-CoV-2 infection. The fold increases in GMT were larger against Omicron sub-lineages compared to against D614G. Increases in omicron sub-lineage neutralization following omicron BTI has been observed in other cohorts [45–47]. Furthermore, boosting mRNA vaccinated mice with an omicron/BA.1 based mRNA vaccine increased serum neutralization activity and protection against omicron/BA.1 [48]. Recent studies indicate that the increase in cross-neutralizing activity is mostly driven by re-activation of B cell clones initially generated through vaccination with Wuhan-1 Spike that cross-react with the VOC, with little evidence of the generation of a *de novo* VOC-specific antibody response [46,49,50]. These observations are contradictory to recent reports suggesting that antibody responses to omicron/BA.1 were limited following an omicron BTI in triple vaccinated donors who had been infected prior to vaccination [51].

The findings of this study are applicable in populations where COVID-19 vaccine coverage remains low and where there is limited vaccine supply. In vaccine naïve populations, an extended interval will likely be beneficial through generation of broader antibody-based immunity against VOCs in the first six months following the 2^nd^ vaccine dose. Indeed, vaccine effectiveness was shown to be significantly higher in those receiving a longer interval between mRNA doses [52,53]. However, whilst there is broader activity following the 2^nd^ vaccine dose in the extended group, there would be a longer period following the 1^st^ vaccine in which antibody titres will remain low and narrow which in turn may lead to a higher risk of infection during this period. This is particularly relevant in vulnerable patient populations, e.g. cancer patients [54–57] and those on immunosuppressive treatment [58,59], where many participants failed to seroconvert following a single vaccine dose. Indeed, UK policy was changed to allow these patient groups a 2^nd^ vaccine dose 3–4 weeks later. Importantly, despite the interval between the 1^st^ and 2^nd^ vaccine doses, a third vaccine dose increases neutralization against the current VOCs including omicron sub-lineages BA.1, BA.2, BA.4 and BA.5. This study does not assess whether the broader neutralization response in the extended-group reduced breakthrough infections. The longer interval in the short-group between 3^rd^ vaccine and BTI is likely associated with the timing of 3^rd^ vaccine dose administration and the UK omicron infection wave. The short-group received a 3^rd^ vaccine in early September 2021 whereas the extended-group received the 3^rd^ dose between November-December 2021.

A limitation of this study is the relatively small sample size in each group which limits the power of the statistical analysis, and it does not allow the assessment of how timing of prior SARS-CoV-2 infection influences antibody responses following vaccination. Furthermore, the impact on cell-mediated immune responses has not been investigated. However, Hall *et al* have previously show that no significant differences in Spike-specific polyfunctional CD4+ T cell responses between the short and extended groups [60] whereas Payne *et al* reported higher levels of these cells in the extended group [30].

In summary, in a SARS-CoV-2 naïve population, an extended interval between the 1^st^ and 2^nd^ doses of BNT162b2 leads to an increased neutralization breadth, Spike binding avidity and frequency of Spike-reactive memory B cells. However, this advantage is lost when a 3^rd^ vaccine dose is administered 6–8 months later where broad neutralization against current omicron sub-lineages is observed in both groups. These findings provide insights into optimizing vaccine regimens to provide the greatest neutralization breadth.

## Materials and methods

### Ethics statement

This study used human samples collected with written consent as part of a study entitled “Antibody responses following COVID-19 vaccination”. Ethical approval was obtained from the King’s College London Infectious Diseases Biobank (IBD) (KDJF-110121) under the terms of the IDB’s ethics permission (REC reference: 19/SC/0232) granted by the South Central–Hampshire B Research Ethics Committee in 2019.

### Protein expression and purification

Recombinant Spike and nucleoprotein for ELISA were expressed and purified as previously described [12,13].

### ELISA (Spike)

96-well plates (Corning, 3690) were coated with antigen at 3 μg/mL overnight at 4°C. The plates were washed (5 times with PBS/0.05% Tween-20, PBS-T), blocked with blocking buffer (5% skimmed milk in PBS-T) for 1 h at room temperature. Serial dilutions of plasma in blocking buffer were added and incubated for 2 hr at room temperature. Plates were washed (5 times with PBS-T) and secondary antibody was added and incubated for 1 hr at room temperature. IgG was detected using Goat-anti-human-Fc-AP (alkaline phosphatase) (1:1,000) (Jackson: 109-055-098). Plates were washed (5 times with PBS-T) and developed with AP substrate (Sigma) and read at 405 nm.

### ELISA to determine previous SARS-CoV-2 infection (Nucleoprotein and Spike)

96-well plates (Corning, 3690) were coated with antigen (Spike or nucleoprotein) at 3 μg/mL overnight at 4°C. The plates were washed (5 times with PBS/0.05% Tween-20, PBS-T), blocked with blocking buffer (5% skimmed milk in PBS-T) for 1 h at room temperature. Plasma was diluted at 1:25 in blocking buffer were added and incubated for 2 hr at room temperature. Plates were washed (5 times with PBS-T) and secondary antibody was added and incubated for 1 hr at room temperature. IgG was detected using Goat-anti-human-Fc-AP (alkaline phosphatase) (1:1,000) (Jackson: 109-055-098). Plates were washed (5 times with PBS-T) and developed with AP substrate (Sigma) and read at 405 nm. As reported previously [12], baseline plasma (extended-group) with OD values 4-fold above background for both IgG to Nucleoprotein and IgG to Spike were considered previously infected with SARS-CoV-2. Where baseline samples were not available (short-group) then IgG to N alone was used to determine a previous SARS-CoV-2 infection.

### Avidity ELISA

The ELISA was carried out as described above. A 4-point titration (starting at 1:25, 1:4 dilution series) was used. After incubation of plasma, one half of the plate was incubated with 8M Urea and the other half incubated with PBS for 10mins before washing 5-time with PBS-T. The area under the curve was determined in Prism (Log dilution). The avidity index was calculated using the following formula:

$$Avidity\;index=100*\left(\frac{Area\;under\;curve\;with\;8M\;Urea}{Area\;under\;curve\;with\;no\;Urea}\right)$$

### Neutralisation assay with SARS-CoV-2 pseudotyped virus

Pseudotyped HIV-1 virus incorporating the SARS-CoV-2 Spike protein (either wuhan-1, D614G, alpha (B.1.1.7), beta (B.1.351), delta (B.1.671.2), mu (B.1.621), omicron (BA.1, BA.2 or BA.4/5) were prepared as previously described [13,61]. Viral particles were produced in a 10 cm dish seeded the day prior with 5x10^6^ HEK293T/17 cells in 10 ml of complete Dulbecco’s Modified Eagle’s Medium (DMEM-C, 10% FBS and 1% Pen/Strep) containing 10% (vol/vol) foetal bovine serum (FBS), 100 IU/ml penicillin and 100 μg/ml streptomycin. Cells were transfected using 90 μg of PEI-Max (1 mg/mL, Polysciences) with: 15μg of HIV-luciferase plasmid, 10 μg of HIV 8.91 gag/pol plasmid and 5 μg of SARS-CoV-2 spike protein plasmid [62,63]. The supernatant was harvested 72 hours post-transfection. Pseudotyped virus particles was filtered through a 0.45μm filter, and stored at -80°C until required.

Serial dilutions of plasma samples (heat inactivated at 56°C for 30mins) were prepared with DMEM media (25μL) (10% FBS and 1% Pen/Strep) and incubated with pseudotyped virus (25μL) for 1-hour at 37°C in half-area 96-well plates. Next, Hela cells stably expressing the ACE2 receptor were added (10,000 cells/25μL per well) and the plates were left for 72 hours. Infection levels were assessed in lysed cells with the Bright-Glo luciferase kit (Promega), using a Victor X3 multilabel reader (Perkin Elmer). Each serum sample was run in duplicate and was measured against the five SARS-CoV-2 variants within the same experiment using the same dilution series.

### FACS analysis of Spike-specific memory B cells

Flow cytometry of cryopreserved PBMCs was performed on a BD FACS Melody as previously described. SARS-CoV-2 Wuhan-1 Spike was pre-complexed with the streptavidin fluorophore (Alexa-488) at a 4:1 molar ratio prior to addition to cells. PBMCs were incubated with Fc block for 15 minutes at 4°C. PBMCs were stained with anti-CD3-BV510 (Biolegend), anti-CD19-PerCP-Cy5.5 (Biolegend), anti-IgD-Pacific Blue (Biolegend), anti-CD27-BV785 (Biolegend), anti-CD38-APC-Cy7 (Biolegend), anti-CD20-APC (Biolegend), anti-IgG-PE-Cy7 (BD Biosciences), anti-IgM-PE (Biolegend) and Spike-Alexa Fluor 488 for 1 hour at 4°C. PBMCs were washed with PBS and stained with live/dead for 1 hour at 4°C.

## Supporting information

S1 Fig SARS-CoV-2 Spike-reactive memory B cells measured before and after vaccination. **A)** Example FACS gating for pre-vaccination sample (baseline) and from post-vaccination sample (post 2^nd^) from SARS-CoV-2 naïve individual. **B)** Frequency of memory B cells in the total B cell population at baseline and post 2^nd^ vaccine for matched donors.
(TIF)
