# Supplementary figures and images for: The effect of Omicron breakthrough infection and extended BNT162b2 booster dosing on neutralization breadth against SARS-CoV-2 variants of concern
Source: PLoS Pathog. 2022 Oct 3;18(10):e1010882. doi: 10.1371/journal.ppat.1010882 (PMC9560610; doi:10.1371/journal.ppat.1010882)

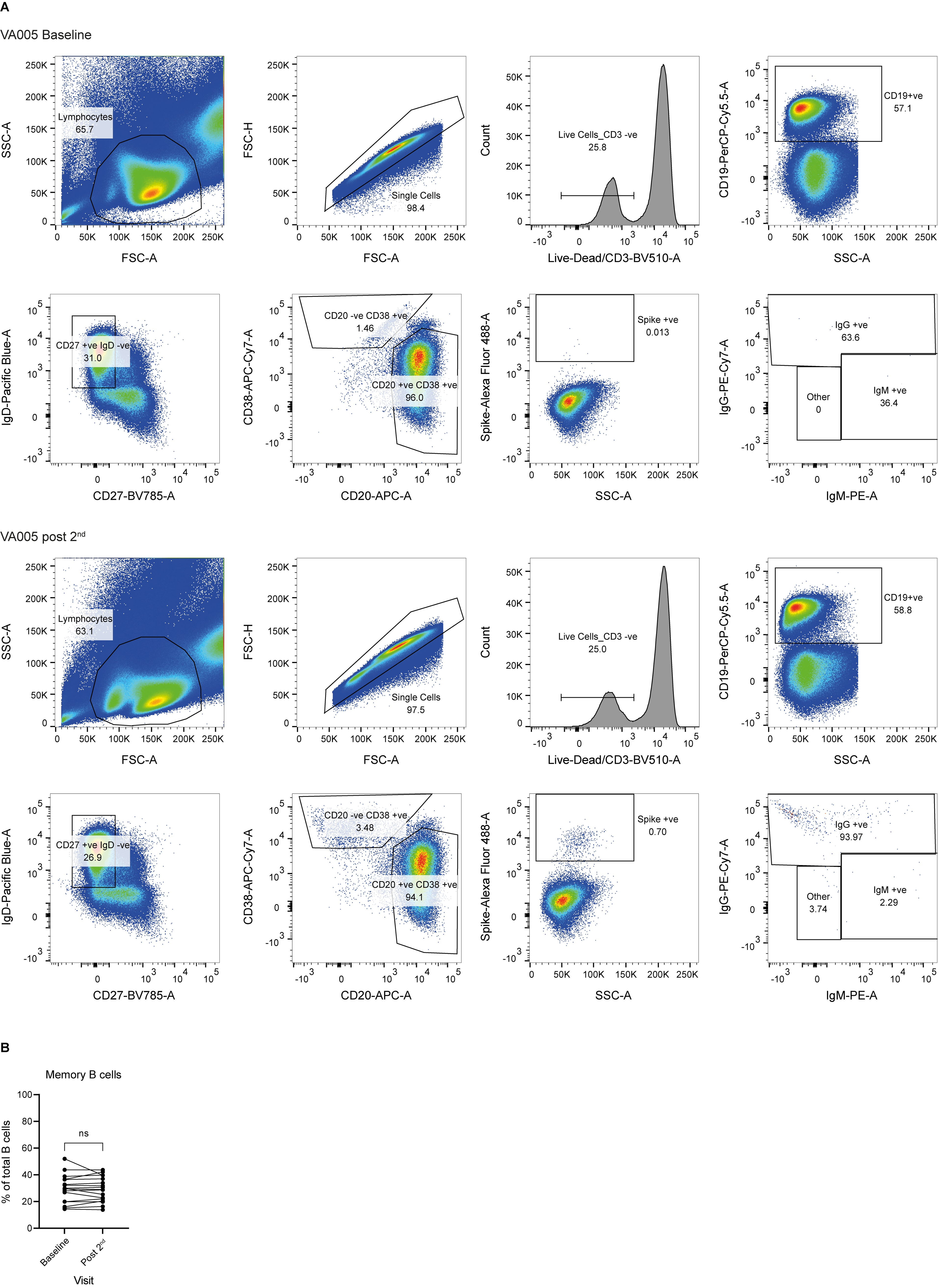

Supplement: S1 Fig — A) Example FACS gating for pre-vaccination sample (baseline) and from post-vaccination sample (post 2nd) from SARS-CoV-2 naïve individual. B) Frequency of memory B cells in the total B cell population at baseline and post 2nd vaccine for matched donors. (TIF) [file ppat.1010882.s001.tif]
